# Sodium Intake and Health: What Should We Recommend Based on the Current Evidence?

**DOI:** 10.3390/nu13093232

**Published:** 2021-09-16

**Authors:** Andrew Mente, Martin O’Donnell, Salim Yusuf

**Affiliations:** 1 Population Health Research Institute, Hamilton Health Sciences, Hamilton, ON L8L 2X2, Canada; martin.odonnell@nuigalway.ie (M.O.); salim.yusuf@phri.ca (S.Y.); 2 Department of Health Research Methods, Evidence and Impact, McMaster University, Hamilton, ON L8S 4L8, Canada; 3 HRB-Clinical Research Facility, National University of Ireland, H91 TK33 Galway, Ireland; 4 Department of Medicine, McMaster University, Hamilton, ON L8S 4L8, Canada

**Keywords:** sodium, salt, blood pressure, cardiovascular disease, mortality, renin–angiotensin–aldosterone system, population health

## Abstract

Several health organizations recommend low sodium intake (below 2.3 g/day, 5.8 g/day of salt) for entire populations, on the premise that lowering of sodium intake, irrespective of its level of intake, will lower blood pressure and, in turn, will result in a lower incidence of cardiovascular disease. These guidelines were developed without effective interventions to achieve long term sodium intakes at low levels in free-living individuals and without high-quality evidence that low sodium intake reduces cardiovascular events (compared with average levels of intake). In this review, we examine whether advice to consume low amounts of sodium is supported by robust evidence. We contend that current evidence indicates that most people around the world consume a moderate range of dietary sodium (3 to 5 g/day), that this level of intake is associated with the lowest risk of cardiovascular disease and mortality, and that the risk of adverse health outcomes increases when sodium intakes exceeds 5 g/day or is below 3 g/day. While the current evidence has limitations, it is reasonable, based upon prospective cohort studies, to suggest a mean target of below 5 g/day in populations, while awaiting the results of large randomized controlled trials of sodium reduction on cardiovascular disease and death.

## 1. Introduction

Several health organizations recommend low sodium intake (<2.3 g/day, ~1 teaspoon of salt) for the entire population [1,2,3], a level that has not been achieved by any modern population in the world. The advice to lower sodium to such low levels is based on the premise that lowering sodium intake, irrespective of its current intake levels, will lower blood pressure, which in turn, will lead to reductions in cardiovascular events and deaths. In this framework, it is assumed that extreme lowering of sodium in entire populations is practical, will be beneficial, and will have no harm [4]. However, there is no evidence that it is feasible to sustainably lower sodium in entire populations to low intake levels, and further, the evidence linking sodium consumption with cardiovascular disease has been inconsistent, with no study reporting lower risk of cardiovascular events with low sodium intake (below 2.3 g/day). Therefore, these recommendations have resulted in considerable controversy about what the optimal level of sodium intake should be for good health.

Sodium is an essential nutrient, meaning that it is required for normal body function and health, and therefore expected to have a physiologic ‘healthy’ range of intake, as with other essential electrolytes [5,6]. Most populations globally (≈95%) consume in the range of 3 to 6 g of sodium daily, which means that the current recommended levels of below 2.3 g/day of sodium by entire populations is well below the range of the majority of the world’s experience [7]. Furthermore, these guidelines were developed without evidence that it would be possible to reduce sodium to such low levels on a prolonged basis in entire populations or that such as strategy will lower cardiovascular events or death. Current evidence from cohort studies suggests a J-shaped relationship between sodium intake and cardiovascular events, as would be expected for an essential nutrient [6,8], and suggests that the lowest risk of death or cardiovascular disease occurs in populations consuming an average sodium intake (3 to 5 g/day) [9,10,11,12,13]. To date, no study (observational or randomized trial) has demonstrated a significantly lower risk of cardiovascular events with low sodium intake (below 2.3 g/day) compared with average intake [14].

**Figure 1:**
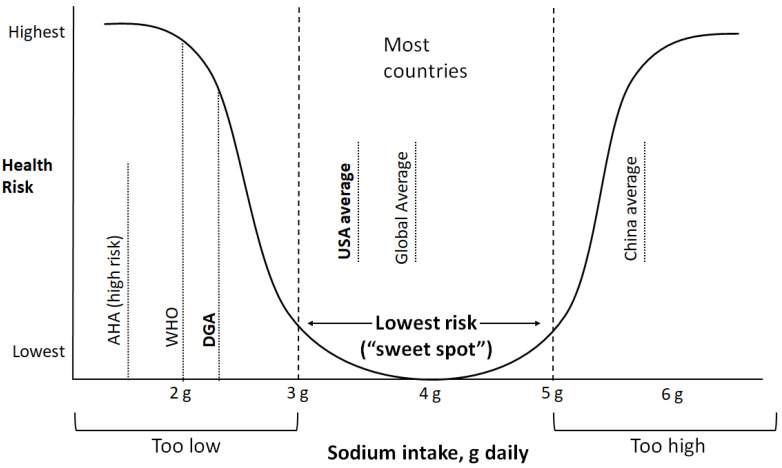
Conceptual diagram of health risk by sodium intake levels based on the current evidence. The lowest risk range (i.e., “sweet spot”) for sodium intake is at ~3 to 5 g/day, with both lower and higher levels of intake associated with higher risk of cardiovascular disease or death. The Dietary Guidelines for Americans (DGA) recommendation for sodium corresponds with a higher risk of adverse health outcomes.

In this review, we assess whether the recommendation for low sodium intake is supported by robust evidence. We contend that current evidence indicates that most populations globally consume a moderate range of dietary sodium (3 to 5 g/day), that this level of intake is associated with the lowest cardiovascular risk (and so is optimal), and that cardiovascular disease risk increases when sodium intakes exceeds 5 g/day or is below 3 g/day (Figure 1).

## 2. Sodium and Physiology

The importance of sodium to human physiology suggests that its relationship with health is likely to have a “sweet spot” (i.e., a J-shaped relationship), meaning that too little or too much is expected to have adverse health consequences [15]. Sodium is the most important extracellular cation in the body, required for many physiologic processes, and is tightly regulated by many processes (renal, biochemical, endocrine, immune, and neural), to maintain blood sodium within a normal range [5,6,16]. Thus, although short-term extreme reductions in sodium intake are possible in controlled settings for short periods, this is unlikely to be sustainable in free living individuals in the long term [17]. In the majority of people with normal kidney function and blood pressure (BP), the kidney is sufficiently able to deal with wide variations in sodium intake, without eliciting increases in BP. However, in some individuals, moderate changes (1 to 2 g/day) in sodium intake can result in marked increases in BP, a concept called salt sensitivity [18]. This can be mitigated by a high potassium diet [19].

Sodium restriction is increasingly shown to activate the renin–angiotensin–aldosterone system (RAAS), which itself is associated with increased cardiovascular risk [20,21]. Two systematic reviews of studies examining sodium restriction and RAAS activity, catecholamines, and lipids reported an increase in renin and aldosterone and catecholamine activity [22,23]. Despite the many available studies (167 in one review), most studies had few participants (usually under 50 individuals), with short duration of follow-up (28 days among people with hypertension, and only 17 days in people without hypertension). Therefore, the sustained long-term effects of low sodium intake on these cardiovascular biomarkers require further study.

Some hunter gatherer populations living in remote settings (e.g., the Amazon Forest) are reported to have a very low sodium intake and low rates of hypertension. However, the measurement of sodium intakes in these populations may not have been accurate, and there is an observed excessive activation of the RAAS system in these populations [24], which may have an adverse effect on health [20,25]. Given their relatively short mean life expectancy (e.g., 40 years), the long-term effects of low sodium intake on health in these populations are unknown [24].

## 3. History of Sodium and Health

It has been theorized that paleolithic humans consumed below 1 g of sodium daily (a claim that is impossible to verify), and that the current mean sodium intake (4 g daily) in human populations is a recent phenomenon (i.e., emerging in the last few millennia) [26]. However, these assertions are unproven, and assume that these people consumed no fish or shellfish, that seawater was not used in food preparation, and that no salt was used to preserve foods. The true amount of sodium consumed by paleolithic humans is unknown, and any estimates are likely wild guesses.

Salt has been instrumental in the transition from hunter gatherer to settled communities, as it allowed the preservation of perishable food during the winter. Salt became an early trading commodity. The word ‘salary’ (from the Latin ‘salarium’) originated as monthly allowance to Roman soldiers to purchase salt, indicating its importance to society. Gandhi stated that “next to water and air, salt is perhaps the most vital to health” [27].

Internationally, the countries with the highest average life expectancy are many of those with the highest average sodium intake [28]. In recent decades, dietary sodium consumption from non-discretionary sources (hidden within foods) has been gradually increasing, particularly in high-income countries [29]. However, sodium intake is tightly regulated through a central neural mechanism, which appears to account for the stable mean sodium intake in populations, despite changes in sodium food sources [5,15].

## 4. Global Sodium Intake

Most people around the world consume a sodium intake in the range of 3 to 6 g/day with fewer than 5–10% consuming below 2.3 g/day, so human experience with the currently recommended low sodium levels is limited [7]. This would also mean that the majority of people in the world will require large changes to their diets to achieve the current recommendations.

Globally, based on a meta-analysis of surveys from 187 countries by the Global Burden of Disease (GBD) collaboration, the mean intake of sodium was estimated at 3.95 g/day [7]. From the INTERMAP study [29], intakes are highest in Eastern Europe, Central Asia, and East Asia (mean intakes above 4.2 g/day). The highest mean intakes were found in the Beijing sample, Northern China, up to 6.9 g/day in men and 5.8 g/day in women. In comparison, in the United States, mean sodium intakes for the eight population samples ranged from 4.1 to 4.4 g/day in men and 3.0 to 3.5 g/day in women.

## 5. Can We Measure Sodium Intake Adequately?

A key challenge in identifying the optimal sodium intake is the lack of a valid and reliable method to objectively quantify sodium intake in a large number of people. The use of multiple 24-h urine collections is sometimes stated to be the reference method, but this approach can be unreliable, as a high proportion of individuals do not comply with the requirement to provide complete urine collections on multiple occasions—a limitation that invalidates this measurement for most studies or assessment of sodium intake in large population studies [30]. (It should be noted that in a recent study by the U.S. Centers for Disease Control, 30% of individuals were not able to provide even a single complete collection of urine) [31].

For population-level studies, given that incidence of cardiovascular events and death in healthy populations are low (about 1% annually), very large sample sizes (several tens or even hundreds of thousands of individuals) are needed to be enrolled and followed for a decade or more to detect modest sized differences in risk for clinical events (e.g., a stroke or heart attack) in those with average vs high sodium intake. Estimating mean intakes in populations is more feasible using a validated calculation formulae from single fasting urine samples (e.g., Kawasaki method with a fasting morning sample) [32,33]. While this approach is not appropriate for assessing sodium intake of individuals in a clinical setting, it has been used for estimating sodium intake in large groups of people epidemiologic studies [11,34]. For example, the PURE study examined sodium intake in over 100,000 individuals based on formula-derived estimates of 24-h urinary sodium excretion from a single fasting urine sample and found a positive association with systolic BP of 2.11 mmHg per g of sodium [11,35], which is consistent with randomized trials of sodium reduction and BP (i.e., 2.42 mmHg reduction in systolic BP per 1 g of sodium lowering) [20] and is stronger than associations reported in observational studies using 24-h urine collections (i.e., 1.0 mmHg change in systolic BP per 1 g of sodium in INTERSALT, 0.22 mm Hg per 1 g in INTERMAP, and ‘null association’ in the Scottish Heart Study) [36,37,38]. These findings strongly indicate that formula-derived estimates of 24-h urinary sodium excretion from fasting urine samples may even be *superior* to capturing ‘usual’ sodium intake than 24-h urine collections for use in large population studies. (Note that the fasting morning urine sample differs from so called “spot urine“ and the distinction is akin to the difference between a fasting blood glucose level vs a random blood glucose value).

## 6. Sodium Intake and Blood Pressure

Current guidelines recommendations for low sodium intake, to under 2.3 g/day, are based solely on studies linking sodium intake with BP in short term intervention trials.

### 6.1. Observational Studies

Overall, there is convincing epidemiologic evidence of a positive, curvilinear association of sodium intake and BP in populations. INTERSALT, the first large international study of sodium and BP, included people aged 20–50 years from 52 centres in 39 countries (*n* = 10,079) who completed a 24-h urine collection. The INTERSALT study [36] reported in 1988 a weak positive association of sodium intake with BP in 33 of 53 centres (which was statistically significant in eight). Another large study in Scotland (*n* = 7354) published at the same time found no association between sodium intake and BP [37], which was confirmed later in the INTERMAP study [38].

The largest international study of sodium intake and BP was the PURE study [35], which included more than 102,000 adults from 18 countries. PURE reported a positive, threshold association of sodium intake with BP (2.11/0.78 mmHg increment in BP per 1 g daily increase in sodium), which was only statistically significant for sodium intakes above 3 g/day and was strongest in those with consumption exceeding 5 g/day (2.58 mmHg increment in BP per 1 g increase in sodium) [35]. The associations were stronger in older people, those with hypertension and those consuming low amounts of potassium. The largest cohort study of sodium intake and BP was the recent UK-Biobank study (*N* = 322,624) [34], which also found higher BP with higher sodium intake.

### 6.2. Randomized Controlled Trials

The impact of sodium intake on BP has been evaluated in numerous clinical trials. Most were short-term trials (95% with less than 6 months duration) with relatively few participants [22,23].

Meta-analyses of clinical trials have found mean reductions in BP with sodium lowering that are generally in keeping with the results from cohort studies [22,23]. In one meta-analysis (36 clinical trials; *n* = 6736), sodium lowering (by an undisclosed amount) was associated with a 3.39/1.54 mmHg reduction in BP, which was greater in people with hypertension than those without hypertension (−4.06/2.26 mmHg vs. −1.38/0.58 mmHg, respectively) [39]. Analyses of the results by differing duration of follow-up reported that the reductions in BP attenuated over time. For example, the between-group differences in BP decreased in a graded fashion with longer follow-up duration (−4.07/1.67 mmHg for the 31 trials of below 3 months duration, −1.91/1.33 mmHg for the five trials of 3–6 months duration, and −0.88/0.45 mmHg for the three trials of more than 6 months duration) [39]. These data demonstrate the near impossibility of achieving and sustaining a low sodium intake (<2.3 g/day) in free-living populations, even with intense dietary counselling.

The DASH-Sodium trial was the largest ‘feeding’ clinical trial of sodium and BP [40] (a feeding trial is one in which all foods that individuals eat are provided for the 30-day duration of the experiment). This was a 3 × 2 factorial trial of 412 participants with pre-hypertension that evaluated three different levels of sodium intake (targeting 1.5, 2.5 and 3.3 g/day) consumed for only *30 days* and compared a DASH diet pattern with a control diet (i.e., an all-around low-quality diet). The BP reduction with sodium lowering was two to three times greater in those consuming the control diet, in which the background potassium intake (1.6 g/day) was well below that of a typical US diet (2.6 g/day) [41], which may have enhanced the effects of sodium reduction on BP in the trial [42]. The findings from the trial have been influential on recommendations to lower sodium intake to below 2.3 g/day and ideally to 1.5 g/day for the entire adult population. However, there are no data that such large reductions in sodium intake would be seen in real-life situations or over a longer period (e.g., in the larger TOHP study where overweight people with borderline elevated BP were counselled to reduce sodium and to eat a healthy diet, the effects over 36 months were very small; systolic BP difference between sodium intake groups was only 1.2 mmHg) [43].

In the TOHP-II trial [43], the largest trial (*n* = 2382) of longer-term sodium reduction and BP (36 months follow-up duration), the mean sodium intake achieved was 3.1 g/day at 18 months and 3.2 g/day at 36 months, despite intensive dietary counselling targeting a sodium intake of <1.8 g/day in the intervention group. This result indicates that a target of <2.3 g/day is not achievable even with intensive efforts to lower sodium in the controlled setting of a clinical trial. The control group had a mean sodium intake of 3.9 g/day at 18 months and 4.0 g/day at 36 months [43].

These studies demonstrate the near impossibility of achieving, and sustaining a low level of sodium intake (below 2.3 g/day) over a few years, in free-living populations, even with intensive dietary counselling.

## 7. Sodium and Cardiovascular Events

### 7.1. Observational Studies

Numerous cohort studies have examined associations of sodium intake with cardiovascular disease and total mortality [44]. Most reviews of these studies compared extremes of sodium intake (i.e., the lowest vs highest intake categories) and assumed a linear relationship [39,45]. By contrast, Graudal et al. [10] evaluated the totality of data, including all levels of sodium intake, and found a J-shaped association of sodium consumption with cardiovascular disease and total mortality, with an increased risk of events both above 5 g/day and below 2.7 g/day, in comparison with moderate levels of intake (2.7 to 5 g/day). These findings were consistent across methods of sodium estimation.

Since the meta-analysis by Graudal, two large prospective studies have been published [11,34], the PURE international study (*n* = 101,945 participants, follow-up of 7.2 years) [11] and the UK-Biobank (*n* = 322,624 participants, follow-up of 7.0 years) [34], both of which employed formula derived estimates of 24-h sodium excretion. The PURE study found a J-shaped association between sodium excretion and cardiovascular disease and total mortality, with lowest risk of events found between 3 and 5 g per day, supporting the previous meta-analysis [10]. The increased risk associated with high sodium intake (above 5 g/d) was largely confined to those with hypertension [11], a finding consistent with the PREVEND study [46]. PURE also showed that in those with high potassium intake and higher-quality diets, the association of high sodium and cardiovascular events was mitigated [47]. The UK-Biobank study reported no significant association between sodium excretion and cardiovascular events, but a suggested J-shaped association was found with mortality [34].

The higher risk for cardiovascular events or death with low sodium intake, compared to average intake, has been seen in studies conducted by several different investigators from over 50 countries [11,12,34,46,47,48,49,50,51,52,53,54,55,56,57,58,59,60] (e.g., PURE [11,53,54], ONTARGET/TRANSCEND [11,51], EPIDREAM [11], EPIC-Norfolk [12], NHANES-I, II and III [55,56,57], FLEMENGHO/EPOGH [50], SURDIAGENE [52], PREVEND [46], and CRIC [58] studies), and has been found in those with and without vascular disease, those with and without diabetes, and those with and without hypertension, and has been observed despite extensive statistical adjustments for confounders and extensive efforts to avoid “reverse causation”. These findings have also been seen in studies using different methods of sodium estimation, including repeated 24-h urine, single 24-h urine, an overnight urine, and dietary assessment.

In 2019, the report by the National Academies of Sciences, Engineering, and Medicine (NASEM) [3] did not take into consideration the evidence from the above cohort studies and meta-analyses that found an increased risk of death or cardiovascular disease events with low sodium intake (J-shaped or inverse association). Instead, the report focused on the observational follow-up data from the control group in the TOHP trial (*n* = 2275 participants; 193 vascular events or deaths) [61]. The NASEM report concluded that sodium intake and cardiovascular disease show a linear association, although there was no significant difference in risk comparing those with low sodium intake (below 2.3 g/day) to moderate sodium intake (3 to 5 g/day), and so therefore the conclusions were not supported by evidence of a statistical difference between these intake groups [61]. In 2017, a technical report from the World Heart Federation, European Society of Hypertension and European Public Health, considered the same information and concluded that the evidence supported sodium-intake reduction only in populations consuming high sodium intake (above 5 g/day), incorporated within a healthy overall diet pattern.

Collectively, there is no robust evidence that lowering sodium below an intake of 3 g/day is likely to lead to a lowering of cardiovascular disease or death compared to a sodium intake of 3 to 5 g/day. There are, however, concerns that sodium intake below 3 g/day may be associated with a *higher* risk of death compared to intakes between 3 and 5 g/day.

### 7.2. Randomized Controlled Trials

Randomized trials to specifically determine the effect of low sodium intake (i.e., below 2.3 g/day) compared to moderate intake on clinical outcomes are still not available.

Meta-analyses of BP trials that reported cardiovascular events have arrived at differing conclusions. One Cochrane collaboration of trials reporting on cardiovascular outcomes concluded that ‘there is insufficient power to confirm clinically important effects’, but reported a 19% lowering of CVD in one analysis of six clinical trials (*n* = 5762) with no significant lowering of mortality [62]. However, this result was dependent entirely on one study [63], a cluster randomized trial with few clusters which may result in poor randomization and bias. Excluding this trial makes the results of the meta-analysis nonsignificant [62].

Importantly, these meta-analyses do not specifically address the impact of low sodium intake (below 2.3 g/day), since the two largest studies in the review (i.e., the observational follow-up of the TOHP-II trial and the trial by Chang et al.), did not achieve the currently recommended target of low sodium intake in the intervention groups. Moreover, the TOHP trial had 23% of participants lost to follow-up for cardiovascular outcomes, but follow-up was complete for mortality–for which no difference in events was observed [64].

Thus, the current evidence is inconclusive as to whether lowering sodium in the diet will reduce CVD, and until we have large clinical trials, the issue will remain unresolved.

### 7.3. Is Sodium Lowering in Populations and Individuals to Recommended Levels Feasible?

Some countries with relatively high mean sodium intakes (above 4 g/day) have shown a lowering of sodium intake. For example, in China, where sodium consumption exceeds that of most countries (above 5 g/day), marked reductions in sodium intake were reported over time (from 6.7 g/day in 1991 to 4.8 g/day in 2009), mainly due to lowering of salt intake at the table or during cooking [65].

By contrast, in countries with more moderate sodium intakes (3 to 4 g/day), there is less clear evidence that population-level interventions result in lowered sodium intake. In the UK, one study reported a reduction in average sodium intake by 0.6 g/day from 2000 to 2011, during which some strategies tailored to lower sodium intake were put into action [66]. However, according to a UK report from 2008, during which time a salt reduction program was fully established, no significant lowering of sodium consumption was reported [67]. Globally, average sodium intake did not decrease during a 20-year period (from 1990 to 2010) [7].

Some guidelines have joint targets for sodium (below 2.3 g/day) and potassium (above 3.5 g/day) intakes [3]. However, since intake of these electrolytes is positively correlated [68], the current combined sodium-potassium target has been nearly impossible to achieve in general populations (less than 0.1% of the population had diets that met the joint guideline target) [47]. In addition, targeting a very low sodium intake may have implications for overall dietary quality. An analysis of the NHANES cohort suggests that achieving an overall high-quality diet is more difficult with sodium intake of below 2.3 g/day than with higher sodium intakes [69].

Overall, we have no evidence that lowering sodium to very low levels (<2.3 g/day) in entire populations is feasible, safe, or beneficial.

## 8. Implications for US Dietary Guidelines

The Dietary Guidelines Advisory Committee (DGAC) classifies sodium as a “nutrient of concern”, based on the belief that Americans consume an excessive amount [70]. While the DGAC states that it “concurs” with the 2013 IOM Report that there is insufficient evidence to support a recommendation of lowering sodium to below 2.3 g/day [71], their conclusions directly contradict the report. In particular, the DGAC does not address the IOM’s conclusion that sodium intake below 2.3 g/day may lead to adverse health outcomes in both healthy and at-risk populations including people with vascular disease and diabetes, a population that includes tens of millions of Americans. Instead, the DGAC recommends that sodium intake among adults should be no more than 2.4 g/day, despite a lack of evidence showing that these amounts are effective and safe, compared to the average (moderate) intake range.

## 9. Conclusions

At present, recommendations to reduce sodium intake in whole populations to low levels is premature. This conclusion was repeated in two recent reviews by a group of experts (with diverse opinions and backgrounds) [14,72]. Of note, the results of several clinical trials of low sodium intake are expected in a year or two. These trials will hopefully clarify the amounts of sodium that are compatible with better health.

We suggest that, until new data emerge (ideally from large clinical trials), the optimal sodium intake should be in the range between 3 and 5 g/day. Most Americans (i.e., about four out of five people) have sodium intakes below 5 g/day, and in these individuals there is little evidence that lowering sodium will reduce cardiovascular events or death. Therefore, efforts to reduce sodium intake in entire populations cannot be justified. A more appropriate strategy would be to use targeted approaches directed at individuals consuming high amounts of sodium (>5 g/day), which, in the US, may also be diets with high intakes of processed foods, where the focus should be on overall health dietary patterns. At-risk individuals, especially the elderly and people with hypertension, it is reasonable to suggest avoiding excessive sodium intake (i.e., >4 g/day), in the absence of orthostatic intolerance syndromes.

Most countries of the world have average intakes within the lowest risk range, with the United States in the lower part of this range (~3.5 g/day). A few countries such as China have average intakes that correspond with higher risk (>5 g/day) and would likely benefit from reducing sodium intake to the “sweet spot” levels.

## Data Availability

Not applicable.
